# Dynamic signatures of the Eureka effect: an EEG study

**DOI:** 10.1093/cercor/bhad150

**Published:** 2023-05-09

**Authors:** Yiqing Lu, Wolf Singer

**Affiliations:** Singer Lab, Ernst Strüngmann Institute for Neuroscience in Cooperation with Max Planck Society, Deutschordenstraße 46, Frankfurt am Main 60528, Germany; Life- and Neurosciences, Frankfurt Institute for Advanced Studies, Ruth-Moufang-Straße 1, Frankfurt am Main 60438, Germany; Department of Neurophysiology, Max Planck Institute for Brain Research, Max-von-Laue-Straße 4, Frankfurt am Main 60438, Germany; Department of Biology, Technische Universität Darmstadt, Schnittspahnstraße 10, Darmstadt 64287, Germany; Singer Lab, Ernst Strüngmann Institute for Neuroscience in Cooperation with Max Planck Society, Deutschordenstraße 46, Frankfurt am Main 60528, Germany; Life- and Neurosciences, Frankfurt Institute for Advanced Studies, Ruth-Moufang-Straße 1, Frankfurt am Main 60438, Germany; Department of Neurophysiology, Max Planck Institute for Brain Research, Max-von-Laue-Straße 4, Frankfurt am Main 60438, Germany

**Keywords:** coherence, dimensionality, Eureka effect, lateralization, phase locking

## Abstract

The Eureka effect refers to the common experience of suddenly solving a problem. Here, we study this effect in a pattern recognition paradigm that requires the segmentation of complex scenes and recognition of objects on the basis of Gestalt rules and prior knowledge. In the experiments, both sensory evidence and prior knowledge were manipulated in order to obtain trials that do or do not converge toward a perceptual solution. Subjects had to detect objects in blurred scenes and indicate recognition with manual responses. Neural dynamics were assessed with high-density Electroencephalography (EEG) recordings. The results show significant changes of neural dynamics with respect to spectral distribution, coherence, phase locking, and fractal dimensionality. The Eureka effect was associated with increased coherence of oscillations in the alpha and theta bands over widely distributed regions of the cortical mantle predominantly in the right hemisphere. This increase in coherence was associated with decreased beta power over parietal and central regions and with decreased alpha power over frontal and occipital areas. In addition, there was a right hemisphere-lateralized reduction of fractal dimensionality. We propose that the Eureka effect requires cooperation of cortical regions involved in working memory, creative thinking, and the control of attention.

## Introduction

Nervous systems need to be able to distinguish activity patterns associated with the search for the solution of a computational problem from those representing a result. This distinction is necessary in order to terminate further search, to eventually convert the result into action, to permit synaptic plasticity for the storage of results, to allow initiation of a new search process, and to permit access of results to awareness. Humans experience solution states as rewarding and are capable of judging the reliability of a particular result. It is pleasant to suddenly arrive at the solution of a perceptual problem, to understand a previously incomprehensible concept, or to solve a puzzle. This sudden transition from searching for a solution to having found the solution has been termed as the Eureka effect (Kaplan and Simon 1990; Sternberg and Davidson 1995; Ahissar and Hochstein 1997; Kounios and Beeman 2014; Sprugnoli et al. 2017). Some of the features of the Eureka experience are probably also shared by higher mammals (Tovee et al. 1996).

However, very little is known about the neuronal underpinnings of the Eureka effect. One of the reasons is that it is hard to predict under which circumstances and exactly when the Eureka effect occurs. Investigations of the Eureka effect are fraught with several methodological problems. First, measurements need to be performed with high temporal resolution because the moment when the Eureka effect occurs is often unpredictable. Second, neurophysiological studies require a sufficient number of comparable trials for analysis.

In the present study, we applied black-and-white Mooney images (Dolan et al. 1997; Tallon-Baudry and Bertrand 1999; McKeeff and Tong 2007; Giovannelli et al. 2010; Castelhano et al. 2013) as the paradigm to induce the Eureka effect. The most interesting feature of Mooney images is that they are initially difficult to interpret but can eventually lead to a stable percept of objects. This percept can also be facilitated by cues, which usually consist of original images (color or greyscale images) of the Mooney images (Hsieh et al. 2010; Goold and Meng 2016). Mooney images have several advantages as test material. First, they permit precise manipulation of parameters such as luminance, contrast, size, and pixels. Second, Mooney images allow the combination of natural objects with complex backgrounds. This permits the induction of strong Eureka effects and the search for neuronal correlates. To maximize the occurrence of Eureka experiences, we developed a method to generate and fine-tune Mooney images that evoke Eureka experiences reliably and reproducibly.

How are ambiguous inputs from Mooney images transformed to unambiguous perceptual solutions? It is suggested that prior experience, stored in memory, is used to modulate peripheral processing in order to facilitate scene segmentation, perceptual grouping, and recognition (Dolan et al. 1997; Gorlin et al. 2012; Goold and Meng 2016). The Bayesian hypothesis of perception posits that internal information, made available by top-down processes, is matched with sensory evidence (Kersten et al. 2004; Friston and Stephan 2007; Clark 2013; de Lange et al. 2018). Thus, the brain is assumed to perform a probabilistic inference that can optimize sensory information to minimize the mismatch between internal and external information. This function can be interpreted as perceptual closure which consists of the completion of incomplete sensory evidence and the correct binding of components into a coherent percept (Grutzner et al. 2010; Moratti et al. 2014). One proposal is that this is achieved through the transient association of distributed neurons into a coherently active assembly (Singer and Gray 1995).

Assuming that the signature of such assemblies is enhanced coherence of neuronal activity (Gray et al. 1989; Singer 1999; Sehatpour et al. 2008; Hipp et al. 2011; Volberg et al. 2013; Singer 2018), we wondered whether, and if so, how the Eureka effect is associated with the changes in neuronal dynamics. We hypothesized that solution states might be associated with enhanced coherence of neuronal activity, as they are likely to result from the successful integration of distributed computational results. To examine this hypothesis, we presented Mooney stimuli to induce the Eureka effect, captured neural activity with high-density EEG recordings, and then investigated how these neuronal responses were modulated during the Eureka effect. The human brain is a highly complex and dynamic system that displays a wide range of state transitions. In light of this, the metrics used in complexity theory are deemed to be valuable indicators of these state transitions and might offer crucial insights into the underlying mechanisms of brain function. In recent years, the utilization of nonlinear methods has gained great interest due to its ability to characterize varying states of both healthy and pathological brain activities (Rodriguez-Bermudez and Garcia-Laencina 2015; Ma et al. 2018; Keshmiri 2020). One of the commonly used metrics is the fractal dimension, a measure of the degree of complexity of a time series, and it has been used to detect changes in the brain’s state during a variety of processes. For instance, it has been used to distinguish between different sleep stages, to identify changes in brain activity associated with the onset of seizures, and as a potential biomarker for various disorders, such as autism, depression, and Alzheimer's disease (Rodriguez-Bermudez and Garcia-Laencina 2015; Ma et al. 2018). For these reasons, we measured not only the power and coherence of oscillatory activity but also measured the fractal dimension of activity vectors (Nikolic et al. 2008; Singer and Lazar 2016) to further characterize the state transitions associated with the Eureka effect.

## Materials and methods

### The selection of images

The original images used for the construction of the stimuli were taken from the Caltech-256 Object Category Dataset (Griffin et al. 2007) and Flickr. More than 30,000 images were included in our initial database. We then manually chose images for further testing according to the following criteria. (i) Familiarity: We chose well known objects, such as animals, plants, furniture, tools, instruments, vehicles, and so on. (ii) Natural and complex background: The images were taken from natural scenes, the target objects being embedded in a complex background, which can render identification difficult.

### The procedure of manipulating the images

The original images were first converted to 256-level greyscale images. As the distribution of greyscale pixels varied widely and was non-normal and as the luminance levels and contrast also differed, adjustments were required. Luminance and contrast were equated with histogram equalization which generates even contrast distributions (Lim 1990).

To create Mooney images with different degradation levels, we applied a frequency-domain Gaussian filter on the greyscale images processed according to the methods described above (Haddad and Akansu 1991). Lowering the cutoff frequency of the filter increases the difficulty to recognize the object in an image. The filters were generated in 3 processing steps: (i) The spatial domain of the original image *f*(*x*, *y*) is transformed to the frequency domain *F*(*u*, *v*) by Fourier transformation; (ii) this *F*(*u*, *v*) is low-pass filtered with a set of cutoff frequencies to obtain a band passed representation *G*(*u*, *v*); and (iii) this representation *G*(*u*, *v*) is then transformed back to the spatial domain *g*(*x*, *y*) (i.e. the blurred image) by an inverse Fourier transformation. In order to obtain images with different degradation levels, the low-pass filter was varied between cutoff frequencies ranging from 10 to 80 Hz (10 Hz per interval) (Supplementary Fig. S1). The median gray level of each image was then chosen as the divider for the assignment of black or white pixels in order to obtain two-tone Mooney images. Subsequently, the recognizability of Mooney images with different cutoff frequencies was tested. This led to the selection of the 20-Hz cutoff frequency for the induction of the Eureka effect because it assured transitions from search to solution most reliably (details are provided in the Supplementary material, see also Supplementary Figs. S2 and S3).

### Participants

In the pilot experiment aimed at the determination of the cutoff frequency, 10 subjects were included. None of these subjects participated in the main experiment (the detailed information of the pilot experiment is given in the Supplementary material). Another 29 healthy subjects (Age 25.2 ± 4.0 years, 16 males, 13 females) took part in the main experiment that comprised both behavioral assessment and EEG recordings. None of these subjects participated in the pilot experiment. Twenty-five subjects were included, and 4 were rejected due to an insufficient number of correct responses. In addition, EEG data from 3 of these 25 subjects had to be excluded from EEG analysis due to insufficient valid data because of artifact rejection. (The artifact rejection is described in the “EEG data preprocessing” section.) All subjects were naive to the experiment, were right-handed, had normal or corrected-to-normal vision, and had no history of neurological or psychiatric disorders. They gave written informed consent before the experiment. The study was approved by the ethical committee of the Goethe University, Frankfurt, and was conducted in accordance with the Declaration of Helsinki. The subjects were recruited from local universities and got paid 15 Euros per hour for their participation.

### Visual stimuli

Presentation (V10.3, Neurobehavioral Systems) was used for stimulus presentation and response collection. All stimuli were generated using Matlab (The Mathworks). The stimuli were displayed as 150 × 150 pixels matrices at the center of a monitor screen with a refresh rate of 60 Hz and were located 70 cm from the subjects’ eye plane, subtending a visual angle of 4.4° × 4.4°, surrounded by gray background, with a gray level of 0.5 on a greyscale of [0, 1].

### The task during EEG recording

**Fig. 1:**
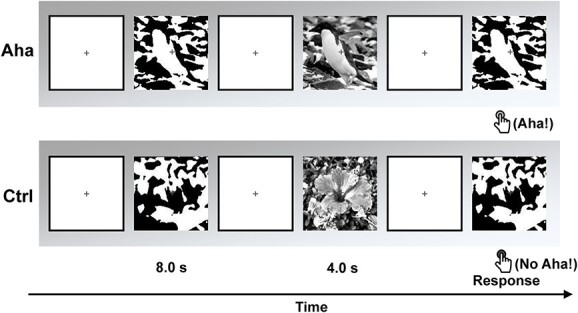
The paradigm during EEG recording. The upper row shows an example of Eureka (Aha) trials which are composed of Mooney images and their congruent greyscale images. The lower row shows an example of control (Ctrl) trials which are composed of Mooney images and incongruent greyscale images.

For each subject, 160 different stimuli were used. At the beginning of each trial, a fixation cross was presented on gray background with a randomized duration between 2 and 3 s. Then, a Mooney image was displayed for 8 s. We address this presentation as the “first stage” of a trial. If the subject could identify the object in the image, they had to press the button “Yes” as soon as possible during this 8-s interval, which terminated the trial. If they could not identify the object in the 8 s, the Mooney image disappeared. Then, a greyscale image was provided as the cue after a random interval of 1.5–2 s. This image lasted for 4 s. In 50% of the trials, the greyscale images were congruent with the last Mooney images and served as cues for the subsequent identification. In the other 50% of the trials, they were incongruent. We address this greyscale image presentation as the “second stage” of a trial. Once the greyscale image had disappeared, the last Mooney image appeared again after a random interval of 2–3 s. We address this repeated Mooney image presentation as the “third stage” of a trial. At this stage, subjects had to press the button “Yes” or “No”, as quickly and accurately as possible, indicating whether or not they could identify the object in the Mooney image. The response needed to be completed within the presentation time of the “third stage” image (i.e. a maximum of 8 s; this time limit was sufficient for subjects to complete the response). If subjects responded with a prompt “Yes” in the matching trials, we took this as evidence that they had experienced a Eureka effect, the sudden recognition of a pattern that they had been unable to identify in the “first stage”. Figure 1 illustrates the task procedure. The order of trials and the order of experimental conditions were randomized. The experiment was evenly divided into 4 blocks. A break of 2–3 min was introduced after each block. Prior to the experiment, a training session with a different set of images was performed in order to allow each subject to practice.

### Data acquisition

The experiment was implemented in an electrically shielded, sound-attenuated, darkroom. Subjects watched the monitor which was outside the room through an electrically shielded window. The EEG was recorded with a HydroCel Geodesic Sensor Net 130 with 128 channels (Electrical Geodesics, Inc.), with the reference electrode at Cz. The electrodes were spaced over the head following the instructions. Electrode impedances were kept <50 kΩ. Data were sampled at 1,000 Hz and were digitally saved on a Mac system for offline analysis.

### Behavioral analysis

We subdivided trials into 2 groups: an Eureka (Aha) group and a control (Ctrl) group. The Aha group consisted of trials (with congruent cue) and “Yes” responses at the “third stage”. The Ctrl group consisted of trials (with incongruent cue) and “No” responses at the “third stage”. Trails that did not meet these requirements (9.5% of trials) were discarded from further analysis. We then compared the distributions of reaction times (RTs) for trials of Aha and Ctrl groups.

### E‌EG data preprocessing

In Net Station software 4.5.1 (Electrical Geodesics, Inc.), the continuous EEG signal was high-pass filtered (0.3 Hz) and notch filtered (50 Hz), and then segmented into a series of 2–10-s long epochs (depends on the RTs). Each trial segment started 1 s before the onset of the “third stage” and ended 1 s after the response during the “third stage.” In the following steps, data were analyzed using the Matlab toolbox FieldTrip (Oostenveld et al. 2011) and our customized scripts. The semiautomatic artifact rejection was supplemented by visual inspection, Fieldtrip scripts, and independent component analysis to detect electrode drifts, eye movements, and electromyographic and electrocardiographic interference (Amari et al. 1995; Bell and Sejnowski 1995; Lee et al. 1999; Anemuller et al. 2003). Rejected channels were interpolated using spherical splines. To avoid contamination of neural activity induced by stimulus onset rather than Eureka effects, only the trials with RTs longer than 0.5 s were analyzed.

### Spectral analysis

To investigate the time-frequency activities that are involved in the Eureka effect, we analyzed the spectral changes of EEG signals over the whole electrode space, using the Hanning taper approach, with a 5-cycle time window and variable width of frequency smoothing depending on the frequency, with 1-Hz steps. The analysis time window was 1 s (for stimulus-locked epochs, from −0.25 to 0.75 s around stimulus presentation; for response-locked epochs, from −0.75 to 0.25 s around the response). For baseline correction, a time period after stimulus onset of the “first stage” was used, which had the same length as the analysis window used in the “third stage”, whose duration was determined by the respective RTs. In summary, the baseline correction and the comparison between Aha and Ctrl can be described as a simple formula: *Diff* = (*M*3_Aha_ − *M*1_Aha_) − (*M*3_Ctrl_ − *M*1_Ctrl_), in which, *Diff* is the difference between Aha and Ctrl; *M*3 and *M*1 refer to data from Mooney images at the “third stage” and the “first stage”, respectively; *M*3 − *M*1 refers to a dB conversion specifically for spectral analysis. The subtraction removes the components resulting from the stimulus-evoked responses. Finally, we calculated grand averages for each condition and participant. The theta, alpha, beta, and gamma bands were defined by the following frequency ranges, respectively: 4–7, 8–12, 13–30, and 31–100 Hz.

In order to avoid the bias that may be introduced by unequal numbers of trials, the number of trials for Aha and Ctrl groups was equalized before comparison by randomly discarding trials (18.9% of trials) from the condition with a larger number of trials. This correction was also performed for coherence and phase locking value (PLV) analysis.

### Coherence

Brain networks are defined both anatomically and functionally. Given the high degree of anatomical connectedness among processing streams, any cognitive and executive task requires fast and flexible formation of functional networks. One suitable measure for functional connectivity is coherence (Rosenberg et al. 1989). It takes into account both phase and amplitude components of signals and provides information about the anatomical and functional coupling of network nodes. We therefore calculated coherence in specific frequency bands in selected time windows between electrode positions/channels. Coherence was calculated according to the formula below:


$$ C(f)=\left|\frac{\sum \limits_k{A}_k(f){B}_k(f){e}^{j\left({\phi}_k(f)-{\theta}_k(f)\right)}}{\sqrt{\sum \limits_k{A}_k{(f)}^2}\sqrt{\sum \limits_k{B}_k{(f)}^2}}\right|, $$

where ${A}_k(f){e}^{j{\phi}_k(f)}$ and ${B}_k(f){e}^{j{\theta}_k(f)}$ describe the Fourier-transformed signals, and *k* is the trial number (Srinath and Ray 2014). The coherence values are real-valued numbers from 0 to 1. One indicates that two signals have perfect coupling, while 0 indicates independence. The coherence values described in the results were all baseline corrected according to the method applied for the spectral analysis. Since standard coherence measures may be affected by volume conduction (Nolte et al. 2004), we also analyzed coherence by using the imaginary part of the coherence (iCOH) value, which is less compromised by volume conduction. The coherence values were calculated for all electrode combinations.

### Phase locking value

As coherence values are sensitive to amplitude variations, we also calculated PLVs. These values reflect phase correlations between two oscillatory signals (Lachaux et al. 1999; Srinath and Ray 2014) and serve as an index for the synchronization of different neuron groups. The key factor distinguishing the PLV from coherence is that the PLV does not take amplitude into account. It only measures the phase component. Similar to coherence, the PLV also has a real-valued number ranging from 0 to 1. The value 1 indicates that two signals are strictly phase locked, while 0 indicates that their phase relations are random. The PLV can be represented as


$$P(f)=\frac{1}{N}\left|\sum \limits_k{e}^{j\left({\phi}_k(f)\kern0.33em -\kern0.33em {\theta}_k(f)\right)}\right|,$$

where *ϕ_k_*(*f*) − *θ_k_*(*f*) is the phase difference between two signals, *N* is the number of trials, and *k* is the trial number. The PLVs described in the results are all baseline corrected according to the method applied for spectral analysis. Similar to the analysis of coherence, we also used the imaginary part of PLV (iPLV) to reduce the potential effects of volume conduction. The PLVs were calculated for all electrode combinations.

### Laterality index

To capture the lateralization effects, we calculated laterality indices (LIs) (Jansen et al. 2006; Wilke and Schmithorst 2006; Seghier 2008). LIs were calculated by evaluating the differences between the right and left hemispheres. We used the following formula to determine the


$$LI = \frac{V_R-{V}_L}{V_R+{V}_L},$$

where *V_R_* and *V_L_* refer to averaged values within the right and left hemispheres, or the right and left electrode clusters. A positive or negative value of LI indicates lateralization of neural activity to the right or left side, respectively. To calculate the LI of coherence and PLV, only a selection of electrodes were used. Right electrodes: Fp2, F4, F8, C4, T4, P4, T6, and O2; left electrodes: Fp1, F7, F3, T3, C3, T5, P3, and O1; midline electrodes: Fz, CPz, Pz, and Oz. The values of coherence and PLV of the right hemisphere were calculated from intra-“right electrodes” combinations and “right electrodes”–“midline electrodes” combinations. Similarly, the values of the left hemisphere were calculated from intra-“left electrodes” combinations and “left electrodes”–“midline electrodes” combinations. The electrode locations were defined according to the 10–20 EEG system.

### Dimensionality

In order to assess changes in the complexity of network dynamics, we determined the fractal dimension of the recorded high-dimensional time series. The EEG data were normalized to *z*-scores and were then analyzed for fractal structure. The principle of the analysis is based on scale-versus-count relationships. For *n* points inside spheres of a certain size *δ* (i.e. the distances between these points are < *δ*),


$$C\left(\delta \right)=\underset{n\to \infty }{\lim}\frac{1}{n^2}\left|({x}_i,{x}_j)\right|,$$

where (*x_i_*, *x_j_*) are pairs of points with the indices of *i* and *j* (|*x_i _ − x_j_* | < *δ*, *i* ≠ *j*), and *C*(*δ*) is the correlation integral (Grassberger and Procaccia 1983). The dimension *D* can be estimated by the slope of ln(*C*(*δ*)) versus ln(*δ*), given by:

$\qquad\qquad\qquad\qquad\qquad D=\ln \left(C\left(\delta \right)\right)/\ln \left(\delta \right).$

Under ideal conditions, the slope is a constant that represents perfect self-similarity. But under real conditions, the slope can change across scales, providing additional information of the multifractals in the data series (Nikolic et al. 2008).

The dimension was calculated for clusters of electrodes that were selected depending on their positions. Ten clusters were formed according to the proposal by Marie and Trainor (2013). The clusters cover the left frontal, left central, left parietal, left temporal, left occipital, right frontal, right central, right parietal, right temporal, and right occipital areas (Figs. 8 and 9). Each cluster included 8–10 electrodes.

### Statistics

To evaluate the significance of differences between trials with the Eureka effect and the control condition, we performed a Wilcoxon signed-rank test when samples were not normally distributed; or a *t*-test (two-tailed) when samples were normally distributed. A cluster-based nonparametric randomization test was used to solve the multiple comparisons problem (Maris and Oostenveld 2007). For each paired sample, an independent samples *t*-test was computed. All samples with a *P*-value lower than a threshold of 0.05 were selected and were then clustered on the basis of spatial and temporal adjacency. The sum of the clustered *t* values was calculated. These samples were then randomized across Aha and Ctrl conditions, and the cluster *t* value was analyzed. This step was repeated for 1,000 times. We then obtained a new distribution of cluster *t* values. On the basis of this distribution, the *t* value from the original data was evaluated. Here, we used the threshold 0.05 for significance and to obtain significant data cluster(s) (for example: including electrodes and time windows for predefined frequencies). Similarly, the cluster-based nonparametric randomization test was also used to correct the *z* value of the Wilcoxon signed-rank test.

## Results

Our study investigated the electrophysiological signatures of EEG-recorded brain activity during the Eureka effect. In this section, we will describe in subsequent paragraphs the behavioral results, changes in power in different frequency bands, changes in coherence and PLVs in the alpha and theta bands, and changes in fractal dimensionality.

### Behavioral data

Subjects continued 83.9% of the trials till the “third stage”, and 90.5% of these trials had correct responses (for congruent trials: 86.0%; and for incongruent trials: 95.5%). Correct meant “Yes” response to congruent trials or “No” responses to incongruent trials. This result suggests that the subjects really experienced the Eureka effect and that the comparison between Aha and Ctrl conditions was valid.

**Fig. 2:**
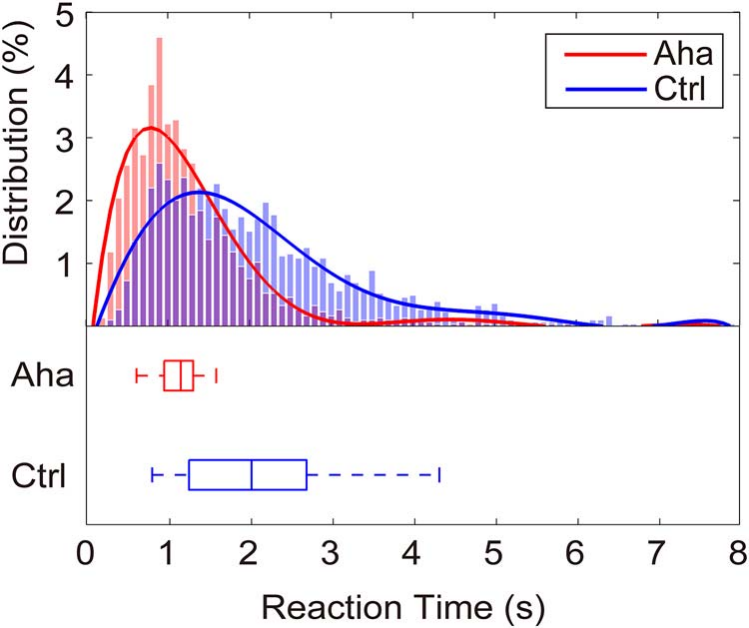
The distribution of RTs. Upper panel: distribution of RTs for all trials. Abscissa: RTs, bin width 0.10 s. Ordinate: frequency of RTs in %. Continuous lines: fitted curves. Lower panel: box plots of the respective RTs distributions, with median values, the 25^th^, and the 75^th^ percentiles. The whiskers indicate extrema.

The RTs of Eureka (Aha) and Control (Ctrl) trials are summarized in Fig. 2. The RTs in Aha trials are significantly faster than the RTs in Ctrl trials (*P* = 5.0699e-109, *Z* = −22.1825, Wilcoxon rank-sum test). The smoothed distribution of RTs shows that the peak of the Aha RTs distribution is at 0.90 s, while the peak of the Ctrl RTs distribution is at 1.50 s. The Aha RTs distribution is narrower than the Ctrl RTs distribution. The median of the Aha trial RTs is 1.13 s and that of the Ctrl trials is 2.00 s. In 92.9% of the Aha trials and 99.3% of the Ctrl trials, the RTs were longer than 0.50 s. These trials were kept for further analysis.

### The spectral changes

#### Alpha power

**Fig. 3:**
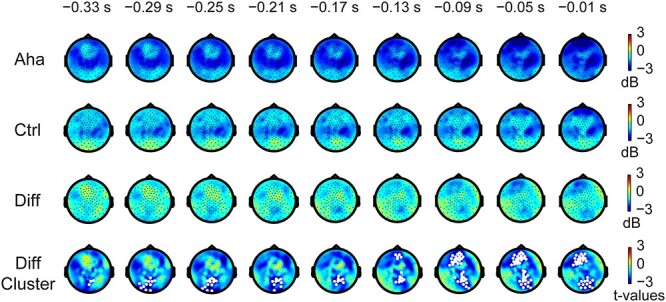
Changes in alpha power before the response to the Eureka-inducing stimulus. The top and second rows: Amplitude (color scale) and distribution of alpha power (baseline corrected) in the Aha and Ctrl conditions at times (indicated above the snapshots) preceding the response. Third row: Difference between Aha and Ctrl (Aha − Ctrl). Fourth row: *t*-values of cluster-based nonparametric randomization test for the difference between Aha and Ctrl. The white regions represent clusters where alpha power decreased significantly (*P* < 0.05).

Given that numerous functions and processes associated with the Eureka effect, such as attention, memory, and top-down modulation, involve activity in the alpha band (Klimesch et al. 2010; Palva and Palva 2011; Clayton et al. 2018), we explored whether alpha power changes in association with the Eureka effect. In stimulus-locked epochs, there was no significant difference in alpha band (8–12 Hz) power between Aha and Ctrl conditions. In response-locked epochs, alpha power was found to be lower for Aha than for Ctrl trials. As shown in Fig. 3 (for a time-frequency plot, see also Supplementary Fig. S4), two clusters (obtained from cluster-based nonparametric randomization tests) of reduced alpha power were found (*P* < 0.05). One was in the frontal region, mostly in the left hemisphere, from 150 ms before response to response (−150 to 0 ms). In this cluster, alpha power was significantly decreased before the response. The second cluster was in the occipital region, from 335 ms before response to response (−335 to 0 ms). Interestingly, this cluster exhibited a gradual shift from the left to the right hemisphere with elapsing time. Before −220 ms, this cluster was mainly located in the left occipital area; after −220 ms, it moved to the right occipital and right superior parietal areas. In brief, we observed decreased alpha power in association with the Eureka effect.

#### Beta power

**Fig. 4:**
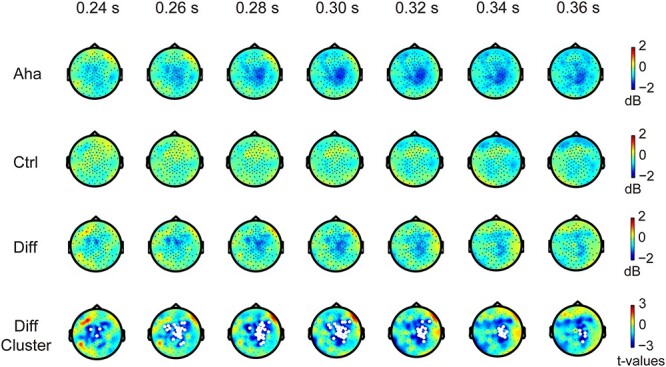
Changes in beta power after stimulus onset. Conventions as in Fig. 3. Note the significant decrease in beta power (white regions in the bottom row) following the Eureka-inducing stimulus.

**Fig. 5:**
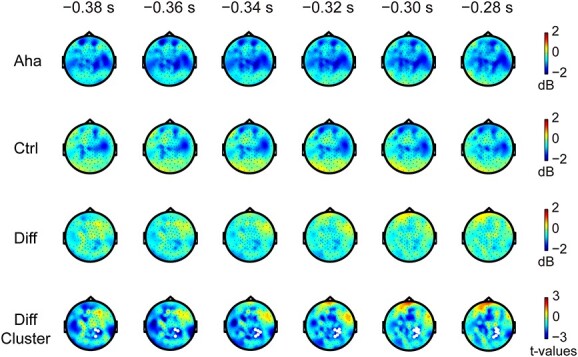
Changes in beta power before the response to the Eureka-inducing stimulus. Conventions as in Figs. 3 and 4. Note the decrease in beta power (white regions in the bottom row; here, *P* = 0.0789) found by cluster-based nonparametric randomization test for the difference between Aha and Ctrl, preceding the response to the Eureka-inducing stimulus.

The Eureka effect involves numerous cognitive processes, and two of them have been linked to the alterations in beta power–memory retrieval and the switching of cognitive states (Sheth et al. 2009; Piantoni et al. 2010). Therefore, we investigated whether beta power changes in association with the Eureka effect. In stimulus-locked epochs, the Eureka effect was associated with a decrease in beta power (13–30 Hz) relative to control in the interval from 240 to 360 ms after stimulus onset (*P* < 0.05), as shown in Fig. 4. This decrease was most prominent in the regions of the right parietal cortex and central gyrus, close to the midline. In response-locked epochs, the cluster-based nonparametric randomization test revealed a strong but not significant trend (*P* = 0.0789) of beta power decrease in the right parietal and right occipital cortices in the time window of −380 to −285 ms before the response (Fig. 5). For a time-frequency plot of decreased beta power, see also Supplementary Fig. S5. In short, the power of beta oscillations decreased in association with the Eureka effect.

#### Theta power and gamma power

Theta band oscillations have been proposed to be involved in various memory processes (Buzsaki and Moser 2013; Roux and Uhlhaas 2014; Herweg et al. 2020) and gamma band oscillations in the integration of sensory evidence with contextual predictions (Singer 1999; Peter et al. 2019). Therefore, we examined whether the power of theta and gamma oscillations changed during the Eureka experience. However, the power analysis of these frequency bands showed no significant differences between the Eureka and control conditions.

### The increase of alpha and theta coherence in the right hemisphere

The spectral power of oscillations is a measure of local synchrony. However, to assess global changes of synchronization, it is necessary to also evaluate the coherence and the PLV of oscillatory activity recorded from different sites. Therefore, we also analyzed coherence and PLV.

#### Alpha coherence

**Fig. 6:**
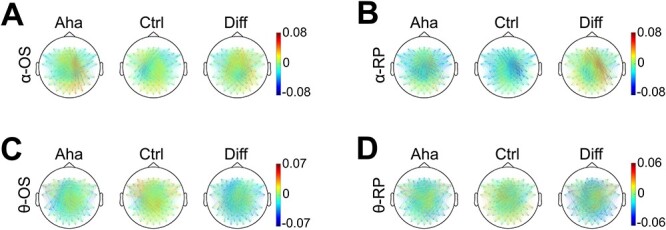
The topographic distribution of A, B) alpha and C, D) theta coherence. Baseline corrected. Diff indicates the difference of values between Aha and Ctrl conditions (Aha − Ctrl). OS represents stimulus onset-locked epochs (A, C) (from stimulus onset to 500 ms after onset). RP represents response-locked epochs (B, D) (from 500 ms before response to response). The colored lines represent the coherence values between electrode pairs (coherence values are indicated by the color scales on the right). Note the different (only for clearer illustration) scales in C and D.

To analyze synchronization between channels in the low-frequency range, we calculated coherence in a 500-ms long window starting at stimulus onset (from 0 to 500 ms, “stimulus-locked epochs”). This analysis revealed an increase of coherence in the alpha band, which was lateralized and particularly prominent in the right hemisphere, for the Aha condition (Fig. 6A). To quantify this lateralization, we calculated the LI of alpha coherence for channel pairs. This confirmed a significant right hemisphere increase of alpha coherence for Aha as compared to Ctrl in the time window from 345 to 450 ms poststimulus (*P* < 0.05). Due to the variable RTs across trials, we also aligned the analysis window to the responses (from −500 ms to response onset, “response-locked epochs”). The right hemisphere-lateralized increase in alpha coherence was present also in this response segment and was significant in the time window from −355 to −275 ms (*P* < 0.05) (Fig. 6B). For the representation of alpha coherence on the timeline, see Supplementary Figs. S6, S9, and S10 (the same applies to the theta coherence). The LI analysis of the iCOH revealed a similar pattern. For stimulus-locked epochs, the right lateralization of increased alpha coherence for Aha versus Ctrl was significant in the time window from 260 to 435 ms (*P* < 0.05) (Supplementary Fig. S7A). For response-locked epochs, the right lateralization was significant in the time window from −410 to −270 ms (*P* < 0.05) (Supplementary Fig. S7B).

#### Theta coherence

Coherence analysis in the theta band also revealed a right-lateralized increase for the Aha condition in the interval of 0–500-ms poststimulus (Fig. 6C). The LI analysis showed that this right lateralization was significant in two time windows: from 125 to 260 ms (*P* < 0.05) and from 375 to 395 ms (*P* < 0.05). For response-locked epochs, there was only a trend for a right hemispheric increase of coherence, but this interhemispheric difference was not significant (Fig. 6D). The iCOH analysis of theta coherence showed that the right lateralization of the Aha-related increase was significant from 180 to 365 ms poststimulus (*P* < 0.05), (Supplementary Fig. S7C). For response-locked epochs, no interhemispheric difference was observed (Supplementary Fig. S7D).

### The increase of alpha and theta phase locking in the right hemisphere

#### Alpha PLV

**Fig. 7:**
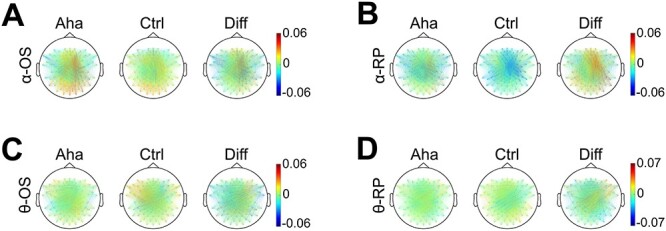
The topographic distribution of alpha and theta PLV. Conventions as in Fig. 6. The colored lines represent the PLV values. Note the different (only for clearer illustration) scale in D. Interestingly, these distributions are similar to coherence in Fig. 6, reflecting that the coherence of alpha and theta bands are associated with phase locking rather than amplitude.

To assess the changes in phase locking associated with the Eureka effect, we analyzed the PLVs for channel pairs in the alpha band from 0 to 500 ms after stimulus onset. Alpha band PLV also increased during the Eureka effect and the topography (Fig. 7A) of this increase resembles that of increases in alpha coherence. The right hemisphere increase of PLVs for Aha as compared to Ctrl was significant within the interval from 385 to 445 ms poststimulus (*P* < 0.05). For the representation of alpha PLV on the timeline, see Supplementary Figs. S6, S11 and S12 (the same applies to the theta PLV). This window overlaps with the interval of enhanced coherence (345–450 ms). PLVs were also enhanced in the response-locked epochs, again more on the right than the left side, but this interhemispheric difference did not reach significance (Fig. 7B). The LI analysis of the iPLV revealed that the right hemisphere increase of iPLVs was significant from 315 to 425 ms poststimulus (*P* < 0.05) (Supplementary Fig. S8A) and from 500 to 395 ms before response (*P* < 0.05) (Supplementary Fig. S8B).

#### Theta PLV

The PLV analysis for stimulus-locked epochs again showed a right-lateralized increase of theta synchrony in the Aha trials (Fig. 7C). The LI analysis indicated that this effect was significant in the time window from 375 to 420 ms (*P* < 0.05). For response-locked epochs, there was also a trend for a right-lateralized increase, but the LI indices did not reach the significance level (Fig. 7D). The iPLVs in the theta band confirmed that the right lateralization was significant from 370 to 405 ms poststimulus (*P* < 0.05), for Aha as compared to Ctrl (Supplementary Fig. S8C). No interhemispheric differences were noted in the response-locked window (Supplementary Fig. S8D).

### Changes in fractal dimensionality

The brain can be considered a complex system exhibiting nonlinear dynamics. Thus, spectral analysis is likely to fall short of capturing the complexity of brain activity associated with switches in cognitive states such as are likely concurrent with the Eureka effect. Therefore, we determined fractal dimensionality, a measure developed to assess the state of complex dynamic systems.

**Fig. 8:**
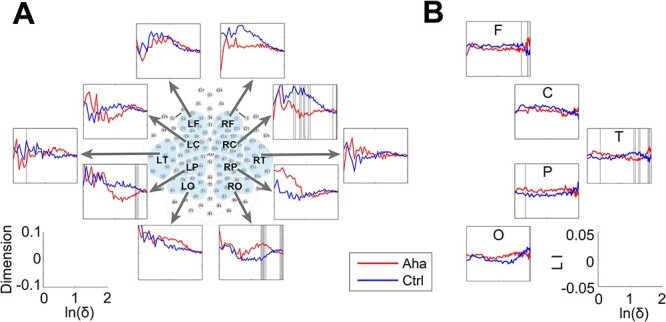
Changes in the dimensionality of activity vectors in the 500-ms interval following stimulus onset. A) Panels representing the dimensionality of activity vectors derived from 10 clusters of recording sites as indicated by the map of electrode coverage (L = left, R = right; F = frontal, C = central, T = temporal, P = parietal, and O = occipital). Baseline corrected. The lower left coordinate indicates the coordinate scale of each subpanel in panel A. Ordinate: dimension; abscissa: natural logarithm of scale size. B) LI of dimension for the clusters. The lower right coordinate indicates the coordinate scale of each subpanel in panel B. Ordinate: LI; abscissa: natural logarithm of scale size. The bins with significant differences between Aha and Ctrl are marked by gray lines.

**Fig. 9:**
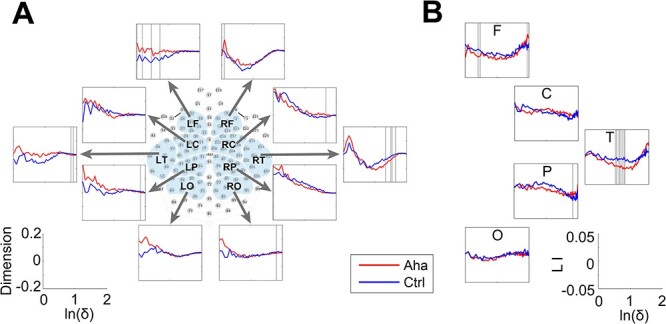
Changes in the dimensionality of activity vectors in the 500-ms interval before the response to the Eureka-inducing stimulus. Conventions as in Fig. 8.

We analyzed dimensionality in the time window from 0 to 500 ms after stimulus onset for 10 predefined clusters of electrodes (described in Materials and methods). We observed significant reductions of dimension over the right central and left parietal areas and a significant increase over the right occipital area (Fig. 8A) in the Eureka trials. Furthermore, LI analysis showed that the dimension reduction was more pronounced (ln(*δ*) from 1.47 to 1.65, *P* < 0.05) in the temporal region of the right hemisphere (Fig. 8B). Similar results were obtained for response-aligned activity patterns. In the time window from −500 ms to the response, there was a significant reduction of dimension over the right temporal area and an increase of dimension over the left frontal area (Fig. 9A). The LI analysis confirmed the right lateralization of the dimension reduction (ln(*δ*) from 0.94 to 1.26, *P* < 0.05) over temporal areas (Fig. 9B).

Taken together, in both the stimulus- and response-locked epochs, the Eureka effect was associated with a reduction of fractal dimension in the right temporal cortex.

## Discussion

We investigated with EEG recordings brain activity during the Eureka effect and found that oscillatory activity in the alpha and theta bands exhibited right hemisphere-lateralized increases in coherence and phase locking, decreased power in the alpha band over frontal and occipital regions, and decreased power in the beta band over right parietal regions and the central gyrus, close to the midline. In addition, we observed a reduction of dimensionality for the activity recorded from the frontal and temporal regions in the right hemisphere. The results of all analyses are summarized in Table 1 in a time resolved manner for stimulus- and response-locked changes.

**Table 1:** A timeline summary of EEG signatures of the Eureka effect in stimulus- or response-locked epochs.

	**Stimulus-locked**	**Response-locked**
Alpha power	N.S.	−150 to 0 ms and −335 to 0 ms^a^
Beta power	240 to 360 ms	−380 to −285 ms^b^
Alpha coherence	345 to 450 ms (260 to 435 ms)^c^	−355 to−275 ms (−410 to −270 ms)
Theta coherence	125 to 260 ms and 375 to 395 ms (180 to 365 ms)	N.S. (N.S.)^d^
Alpha PLV	385 to 445 ms (315 to 425 ms)	N.S. (−500 to −395 ms)^d^
Theta PLV	375 to 420 ms (370 to 405 ms)	N.S. (N.S.)^d^
Dimension	ln(δ): 1.47 to 1.65^e^	ln(δ): 0.94 to 1.26^e^

N.S., not significant.

^a^The first cluster is in the frontal region, from 150 ms before the response to response onset; the second cluster is in the occipital region, from 335 ms before the response to response onset.

^b^ *P* = 0.0789.

^c^The results obtained from the imaginary part of coherence (or PLV) analysis are indicated in parentheses, the same convention for the other frequency bands.

^d^For coherence and PLV, the laterality index analyses do not always reach statistical significance in response-locked epochs, but the trend of increasing coherence and PLV on the right hemisphere is preserved.

^e^For dimension analysis, the logarithmic scale values are obtained from the entire 500-ms interval after stimulus onset or before response.

### Methodological considerations

A crucial question is whether our paradigm reliably elicited a Eureka effect. As reviewed in the introduction, the identification of neuronal correlates of the Eureka effect is hampered by the inability to precisely predict the time of its occurrence and to obtain sufficient trials per subject. Therefore, most of the classical conditions inducing Eureka effects are not suitable for neurobiological investigations. We have settled for a psychophysical task that allowed us to predict with some confidence, whether and when a stimulus would elicit a Eureka experience, and to confirm its occurrence with a behavioral response. Subjects had to accomplish a difficult pattern completion task with physically identical stimuli that required flexible binding of features into a coherent gestalt and could be solved only in a minority of trials despite long inspection time when no additional cues were provided. However, by supplying additional associated information, subjects were enabled to solve the task promptly in the majority of trials, reporting sudden perceptual insight into the solution of the pattern completion problem (Dolan et al. 1997; Giovannelli et al. 2010; Ludmer et al. 2011). The main focus of this study is on the neural processes that are associated with this subjective experience. Our experimental design implied that subjects had to keep in working memory the greyscale images and had to match these templates with the Mooney test stimuli in order to achieve perceptual closure and experience the Eureka effect. In other words, they had to engage top-down processes to resolve the perceptual problem. We carefully titrated the difficulty of the task in the preceding psychophysical experiments in order to assure that our task was distinct from a simple pattern recognition task. During the exposure to the Mooney images at the “third stage”, subjects had to engage several cognitive operations: retrieval of information from working memory, reinterpretation of sparse and ambiguous sensory evidence on the basis of prior knowledge, and identifying the result of this “creative” operation as a solution, the latter being equated with “deeper understanding”. In this respect, our task shared a number of features, with conditions leading to Eureka experiences (see Introduction). Debriefing after the experiment confirmed that subjects indeed experienced a Eureka feeling at the “third stage” of the trials in which they suddenly succeeded to identify the image. We are thus confident that the paradigm allowed for the comparison of neuronal responses to physically identical stimuli that did or did not induce the Eureka effect. In addition, our design allowed us to run a large number of trials on the same subject, which is a prerequisite for neurophysiological studies.

There may be a concern that subjects misclassified recognized objects because they were not asked to name the perceived object. We consider this possibility rather unlikely because the greyscale images were relatively easy to identify. This has been verified in the preceding pilot experiments: The identification rate for the greyscale images was 98.2%. Moreover, even if such misidentifications had occurred, these would not have invalidated the results because subjects would still have experienced an Eureka effect.

Although our paradigm allowed narrowing the time interval during which the Eureka effect was bound to occur, response latencies were still variable. This could have been due to variable latencies of the Eureka effect or to variable lag times between the subjective Eureka experience and the motor response. In order to capture the electrophysiological signatures of the Eureka state, we aligned neuronal responses for analysis to both stimulus and response onset, respectively. By adjusting the duration of the two analysis windows, we made sure to cover the whole interval during which the Eureka effect was bound to occur. Because the results obtained for the two analysis windows were rather similar and located the effect to overlapping epochs between stimulus and response onset, we are confident that the electrophysiological changes reflect processes associated with the Eureka experience.

Coherence analyses based on EEG recordings are fraught with possible confounds resulting from volume conduction. We tried to minimize this problem in several ways: First, we used signals only from widely separated electrodes (*n* = 20) according to the low-resolution 10–20 EEG system (as mentioned in Materials and methods). This allowed resolving hemispheric differences, suggesting sufficient independence of recording sites. Second, we could rely on the “first stage” to control for task independent effects mediated by volume conduction: The use of the “first stage” as baseline for the “third stage” should have reduced the contribution of volume conduction. Third, the adoption of the “imaginary part” approach for the analysis of coherence and PLV (Nolte et al. 2004), which also reduces the effects of volume conduction, gave similar results as the conventional analyses.

### Right hemisphere-lateralized alpha and theta activity

Our data indicate that the Eureka effect is associated with distinct lateralized changes of coherence and power of oscillations in distinct frequency bands.

The most significant correlates of the Eureka effect were enhanced coherence and phase locking of alpha and theta oscillations over the right hemisphere. This enhanced synchronization was not associated with amplitude changes but was only apparent in the measures applied to sensor pairs. This indicates that coherence increases were due to improved phase locking rather than the enhanced amplitude of oscillatory activity in the two frequency bands.

Alpha oscillations have been associated with many different functions. The fact that they tend to be suppressed when neuronal circuits engage in information processing and enter in a regime of high-frequency oscillations has been taken as an indication that they represent an idling rhythm (Pfurtscheller et al. 1996). However, there are also abundant indications for an involvement of alpha oscillations in active processing. Alpha oscillations have been suggested to serve the suppression of irrelevant information in tasks requiring focusing attention (Clarke et al. 2008; Zanto et al. 2010; Zanto et al. 2011; Klimesch 2012; Clayton et al. 2018), to coordinate widely distributed processes by serving as carrier frequency for the establishment of coherence across various frequency bands (cross-frequency coupling) (Palva et al. 2005; Canolty and Knight 2010; Jensen et al. 2014), to be involved in the maintenance of contents in working memory (Palva et al. 2005; Freunberger et al. 2009; Zanto et al. 2014; Foster et al. 2017; Riddle et al. 2020), and to mediate top-down control (Klimesch et al. 2010; Benedek et al. 2011; Palva and Palva 2011; Samaha et al. 2015; Clayton et al. 2018). As elaborated further down, we suggest that the association of increased alpha coherence and phase locking with the Eureka effect might be related to the maintenance and readout of contents from working memory and the top-down mediation of this information to facilitate scene segmentation and perceptual binding.

Theta band activities have been proposed to be involved in various memory processes such as memory encoding, maintenance, and retrieval (Sauseng et al. 2010; Fell and Axmacher 2011; Watrous et al. 2013; Burke et al. 2014; Roux and Uhlhaas 2014; Herweg et al. 2020). For example, investigators have observed increased theta phase locking (Liebe et al. 2012) and increased theta coherence (Sarnthein et al. 1998) between prefrontal and posterior recording sites in working memory retention. Recently, increased theta phase locking has been found connecting a large set of brain regions to support memory encoding and recall (Burke et al. 2013; Clouter et al. 2017; Solomon et al. 2017, 2019; Wang et al. 2018). In the present study, the prior experience stored in memory must be retrieved and used for perceptual grouping and recognition. We thus suggest that the increased theta coherence and phase locking are involved in the maintenance and retrieval of working memory contents required for the solution of our experimental task.

There is a hypothesis that hemispheric asymmetry serves as the underlying structural and functional basis for the Eureka effect (or called insight in some studies), with the right hemisphere playing a prominent role (Kounios and Beeman 2014; Sprugnoli et al. 2017). This hypothesis has been supported by several investigations (e.g. Jung-Beeman et al. 2004; Grabner et al. 2007; Sandkuhler and Bhattacharya 2008; Zhao et al. 2014; Aberg et al. 2017). But it is worth noting that most of these studies have focused on the activity of anatomically specified regions and not on networks. Therefore, more studies (as our study) on the latter are necessary. Moreover, most of these studies used verbal/semantic tasks to elicit the Eureka effect and only a few used visual tasks (e.g. Freunberger et al. 2008). This raises the question why visual tasks generate right-lateralized brain activity like verbal/semantic tasks. One possibility is that (part of) the right hemispheric activity was related to covert verbalization even though subjects were not asked to name the recognized objects. Another possibility is that all these tasks involve creativity (a nondissociable feature of the Eureka effect), which *per se* could lead to an increase in right hemispheric activity. Similar electrographic signatures have been described in previous studies on creativity (Razoumnikova 2000; Grabner et al. 2007; Sandkuhler and Bhattacharya 2008; Kounios and Beeman 2014).

### Dissociation between alpha power and alpha phase locking

Somewhat, unexpectedly, there was a dissociation between alpha power and alpha phase locking. While alpha power decreased, coherence and phase locking increased. This excludes that enhanced coherence was simply a consequence of increased power and suggests nontrivial relations between the power of oscillatory activity and pair-wise synchrony. Power increases in oscillatory population responses can have two reasons. First, an increase in the number of neurons participating in the rhythmic activity, and second, an enhanced precision of synchronization as this enhances the effective summation of currents. In the present case, we observed an enhanced precision of synchronization as reflected by the enhanced coherence and improved phase locking. This suggests that the Eureka effect was associated with a reduction of neurons engaged in alpha oscillations, but, at the same time, with enhanced synchronization of the neuron populations participating in a right hemisphere network engaged in alpha oscillations. A similar dissociation has been described by Freunberger et al. (2008) in an object recognition task with distorted color pictures. Associated with the recognition of the objects, alpha power (9–13 Hz) decreased in occipital and right centro-temporal areas, while phase locking (10–12 Hz) increased in a right hemispheric long-range anterior-to-posterior network. In a later study on working memory, Freunberger et al. (2009) found in posterior areas (parietal-occipital) reduced alpha power and, at the same time, enhanced phase locking. These observations add to the notion that alpha oscillations reflect heterogeneous processes. In line with the idling hypothesis (Pfurtscheller et al. 1996), the decrease in alpha power could reflect the increased engagement of right hemispheric networks and the simultaneous increase in synchronization the emergence of a specific, widely distributed, but sparse network. The latter could serve the mediation of the top-down information required for the memory based perceptual closure of the Mooney images. It remains an open question, whether the alpha and theta networks serve themselves as carrier of information or whether they provide the carrier frequencies for the coordination of other processes (Palva et al. 2005; Canolty and Knight 2010; Jensen et al. 2014; Esghaei et al. 2022).

### Right hemisphere-lateralized reduction of dimensionality

As discussed above, the Eureka effect is associated with the transient formation of large but sparse right hemispheric networks oscillating in the alpha and theta frequency range. This agrees with the dimensionality reduction observed over the right hemisphere.

Dimensionality is a measure of the complexity of dynamic states. If the dimensionality of network activity is high, coding space and degrees of freedom are large but so is ambiguity. A reduction of dimensionality can be interpreted as a reduction of the number of possible states and hence as a reduction of ambiguity (Nikolic et al. 2008; Singer and Lazar 2016). In the present experiments, changes in dimensionality were in general variable and reached significance only rarely. However, there was a robust reduction of dimensionality over the frontal and temporal areas of the right hemisphere. This agrees with the finding that the “solution” state leading to the Eureka experience was associated with enhanced coherence in the alpha and theta frequency bands, again over the right hemisphere. We interpret these changes as an indication that large cortical networks, in particular in the right hemisphere, converged toward a state identified as a solution state due to enhanced coherence and reduced variability.

### Decrease of beta activity

A transient decrease of beta activity has been reported in association with the transition from the maintenance of posture to movement initiation (Schoffelen et al. 2008; Pogosyan et al. 2009; Allen and MacKinnon 2010; Kilavik et al. 2013; Armstrong et al. 2018; Wessel 2020), but it is unlikely that the observed decrease was related to the motor response of our subjects because it should have been canceled by the subtraction of the control condition. A more likely interpretation is that the beta decrease is related to memory retrieval and the switching of cognitive states. The present task required retrieval of information from memory (Sheth et al. 2009; Kounios and Beeman 2014; Sprugnoli et al. 2017), and engaging memory has been found to be associated with decreased beta power in the parietal and parieto-occipital areas (Pesonen et al. 2007; Sheth et al. 2009)**.** As described in the study of Sheth et al. (2009), beta power also decreased in the parietal, parieto-occipital, and centro-temporal areas in subjects solving verbal puzzles. Beta power also decreases transiently with switches in cognitive states (Okazaki et al. 2008; Engel and Fries 2010; Piantoni et al. 2010) and with the disambiguation of visual stimuli (Minami et al. 2014). As sudden switches in cognitive states are a hallmark of the Eureka effect, we propose that the beta decrease observed in conjunction with the Eureka effect is related to such switches. To which extent the decrease in beta power is correlated to changes in neuronal activation cannot be inferred from the EEG recordings. Two studies assessing neuronal activity with positron emission tomography (PET) and functional magnetic resonance imaging (fMRI) (Dolan et al. 1997; Eger et al. 2007) described an increase in activation in the parietal cortex associated with the Eureka experience. How this evidence relates to the transient decrease in beta power is unclear. As changes in neuronal dynamics, such as increases and decreases of synchrony in particular frequency bands, can occur without major changes in average firing rates, there need not be a conflict of these fMRI findings with our present results, both observations actually suggesting an involvement of the parietal cortex in the Eureka effect.

### The Eureka effect and gamma band oscillations

Although the processing of Mooney images, or in a more general context, perceptual closure has been shown to be associated with enhanced power and phase synchronization of gamma oscillations (Uhlhaas et al. 2006, 2009; Castelhano et al. 2013; Moratti et al. 2014), our data did not reveal any significant changes in the gamma band in the Eureka condition. This was somewhat unexpected as gamma synchronization is associated with low-level binding operations (Singer 1999) and successful matching of sensory evidence with contextual predictions in early visual areas (Peter et al. 2019). The solution of the detection task in our experiments did also require the integration of sensory evidence with stored information. However, it is likely that these matching processes occurred at higher levels of the visual processing stream where oscillation frequencies are typically lower than in V1. Moreover, in EEG recordings, the high-frequency oscillations in the gamma frequency range are detectable only when large assemblies of neurons get entrained in sustained, synchronized oscillations. Such entrainment can be achieved in early visual cortex with stimuli that exhibit a high degree of redundancy and regularity in feature space such as high contrast drifting gratings. Cluttered scene stimuli as used in the present experiments are not well suited to induce synchronized gamma oscillations; for a review, see Singer (2021).

## Conclusion

Our data indicate that the Eureka effect involves primarily the right hemisphere in our sample of right-handed subjects. We interpret the increased coherence in the alpha and theta bands as indicators of the formation of widely distributed networks of cortical areas involved in the comparison of sensory evidence with information stored in working memory. The finding that enhanced coherence was associated with a reduction of dimensionality of the dynamic state but not with an increase in power in the respective frequency bands suggests, as neuronal correlate of the Eureka experiences the convergence of a large network to a dynamic state, which is characterized by reduced dimensionality, reduced degrees of freedom and hence reduced ambiguity.

## Abbreviations

Aha, the Eureka (Aha) effect; Ctrl, control; Diff, the difference between Aha and Ctrl (Aha minus Ctrl); EEG, Electroencephalography; fMRI, functional magnetic resonance imaging; iCOH, imaginary part of coherence; iPLV, imaginary part of phase locking value; LI, laterality index; PET, positron emission tomography; PLV, phase locking value; RTs, reaction times.

## Supplementary Material

Supplementary_Material-202304-Lu_bhad150

## Data Availability

The data used in this study are available via a reasonable request to the corresponding author upon signing a formal data sharing agreement. Codes used for this study are open to access at https://github.com/y-q-l/Mooney_EEG.
